# Characteristics of aerobic vaginitis among women in Xi’an district: a hospital-based study

**DOI:** 10.1186/s12905-020-00997-5

**Published:** 2020-06-30

**Authors:** Taohong Zhang, Yan Xue, Ting Yue, Lili Xiong, Xiaowei Wang, Weihong Wang, Ying Liu, Ruifang An

**Affiliations:** 1 Department of Obstetrics and Gynecology, the First Affiliated Hospital of Xi’an Jiaotong University, No.277, Yanta Western Road, Xi’an, 710061 Shaanxi Province China; 2 Department of Obstetrics and Gynecology, Shaanxi Provincial People’s Hospital, Xi’an, 710068 Shaanxi China

**Keywords:** Aerobic vaginitis, Vaginal flora, Mixed infection

## Abstract

**Background:**

Aerobic vaginitis (AV) is a reproductive tract infection that affects health of women. The objective of this study was to analyze the characteristics of simple and mixed AV patients in Xi’an district and provide reference data for the clinical treatment of AV.

**Methods:**

Patients were recruited from the outpatient Department of Obstetrics and Gynecology in the First Affiliated Hospital of Xi’an Jiaotong University from September 2014 to April 2019 in strict accordance with inclusion and exclusion criteria. The study principally examined the vaginal ecosystem, age distribution, levels of functional enzymes, and changes in pH levels in these women. Differences within groups were analyzed.

**Results:**

A total of 284 AV patients were enrolled to investigate the distribution of simple and mixed AV infection. AV infection was found to be mainly simple infection. Simple AV patients were generally aged 50–60 years, while mixed AV patients were mostly aged 30–40 years. In the present study, the density of vaginal bacteria (OR = 13.294, 95% CI = 5.869–30.115, *P* < 0.01), the type of predominant bacteria (OR = 3.962, 95% CI = 1.785–7.984, *P* < 0.01) and positive expression of coagulase (OR = 3.789, 95% CI = 1.798–7.984, *P* < 0.01) were considered risk factors for mixed AV infection.

**Conclusions:**

The epidemiology of simple and mixed AV infection were found to be different, with density of vaginal bacteria (I or IV), species that are predominant and levels of coagulase being risk factors for mixed AV infection.

## Background

Human vaginal flora is a complex and protective environment, which enables the maintenance of vaginal pH levels and microbial balance to withstand the invasion of pathogenic fungi and protozoa. However, any imbalance in the naturally occurring bacterial flora may result in infections such as vulvovaginal candidiasis (VVC), bacterial vaginitis (BV), cytolytic vaginosis (CV) or aerobic vaginitis (AV), the clinical symptoms of which, such as abnormal leucorrhea, increased discharge, vulval itching, burning pain, *and so on,* occurring [1, 2]. Women suffering from these conditions can experience preterm premature rupture of membranes, preterm labor, amniotic fluid infection, chorioamnionitis, sexually transmitted infections, and cervical intraepithelial neoplasia disease [3–6].

AV was first identified by Donders and co-workers in 2002 [7, 8]. In contrast to BV, the prevalence of AV is 7–12%, less prevalent than BV [4]. AV can cause various vaginal symptoms, including stinging and dyspareunia, purulent discharge with significant inflammation and epithelial disruption, a thick cottage-cheese-like discharge associated with vaginal and vulvar pruritus, pain, burning, erythema, and edema. Secondary infections may also occur, the outcomes of which can be serious, including miscarriage, chorioamnionitis, premature rupture of membranes, preterm delivery, infertility, and pelvic inflammatory disease (PID) [9]. However, the pathogenesis of AV remains unclear although it is under investigation. A preview study indicated that it resulted from an immunological response with increased inflammatory reaction [10]. In addition, the microflora in AV comprises commensal aerobic microorganisms of intestinal origin, principally *Escherichia coli*, *Staphylococcus aureus* and coagulase-negative Staphylococci [7, 11]. A recent study indicated that being unmarried, use of an intrauterine device, long-term use of antibiotics, and frequent vaginal douching were risk factors for AV [12].

The principal diagnostic technique for identification of AV is currently observation of a wet film of vaginal secretion under a light microscope, combined with clinical manifestations. However, assessment of a wet film can lead to errors due to the subjective judgment of clinicians. In addition, results of diagnosis vary across different hospitals, regions and even countries depending on the levels of knowledge and skills of investigators. Therefore, this study aimed to investigate the characteristics of distribution of AV in patients, the levels of vaginal enzymes and pH values, to explore the use of particular vaginal enzymes in diagnosis, and provide reference data for the reliable and objective diagnosis of AV in the future.

## Methods

### Subjects

A retrospective study was conducted in the Gynecology Outpatient Clinic of the First Affiliated Hospital of Xi’an Jiaotong University from September 2014 to April 2019. Women who had symptoms, such as increased discharge, itching of the vulva, burning pain, *etc,* were recruited to the study, in strict accordance with the following inclusion and exclusion criteria. Inclusion criteria: (1) women with a history of sexual activity aged 18 to 50 years old; (2) of Han ethnicity; (3) diagnosis of simple AV infection or AV combined with VVC, BV, TV or others, according to the diagnostic criteria of vaginal infections by microscopic examination and functional enzyme testing; (4) women not menstruating within the previous 3 days, no vaginal irrigation or drug the vagina within the previous 3 days, and no sexual intercourse for 3 days prior to examination. Exclusion criteria were: (1) women who had undergone hysterectomy with or without bilateral excision of the ovary or who had had a genital malignancy; (2) women without test results for enzyme activity or microscopic test of vaginal secretion; (3) women who were pregnant, menstruating or lactating; (4) women who had undergone chemotherapy, radiotherapy or hormonotherapy due to a malignancy. Before recruitment to the study, informed consent was obtained from each patient. And the study protocol was approved by the ethics committee of the First Affiliated Hospital of Xi’an Jiaotong University.

### Sampling and tests

The bladder lithotomy position was used for gynecological examination and collection of vaginal discharge from patients who had symptoms such as increased discharge, vulval itching, burning pain, etc. Two vaginal smears were obtained from the upper 1/3 of the lateral vaginal wall for microbiological analysis using standard microbiological methods, analysis of pH value of the vaginal environment, amino acid odor testing, and evaluation of enzymes using a diagnostic strip set for vaginitis (Chaoshi-Bio, Jiangsu, China).

### Diagnostic criteria

(1) AV: Common criteria employed for the diagnosis of AV in patients is Donders’ score or Tempera evaluation [13], but they lack standardization or recognized criteria. However, in accordance with the Chinese expert consensus on the clinical application of vaginal microecological assessment from 2016, AV was assessed and diagnosed using Donders’ score in the present study [14]. Diagnostic criteria and the corresponding AV scores are displayed in Table 1. Scores of 0 to 10 were assigned to each sample, representing different levels of bacterial flora, epithelial disruption, and inflammation: 0–2 (no AV), 3–4 (mild AV), 5–6 (moderate AV), or 7–10 (severe AV). (2) BV: BV was diagnosed using Nugent scoring, in accordance with the Chinese expert consensus on the clinical application of vaginal microecological assessment from 2016 [14, 15]. A score of < 4 was normal, 4–6 moderate, and > 6 was defined as BV. (3) VVC: VVC was diagnosed when hyphae or spores were observed in 10% KOH wet microscopy. (4) Trichomonas Vaginitis(TV): Active trichomonas observed using light microscopy. (5) CV: Cibley diagnostic criteria were used [16].

**Table 1:** Criteria diagnosis of aerobic vaginitis by Donders’ score

AV score	lactobacillary grade	Number of leukocytes	Proportion of toxic leukocytes	Background flora	Proportion of parabasal epithelial cells
0	I and IIa	≤10/hpf	None or sporadic	Unremarkable or cytolysis	None or < 1%
1	IIb	> 10/hpf and ≤ 10/epithelial cell	≤50% of leukocytes	Small bacilli	≤10%
2	III	> 10/epithelial cell	> 50% of leukocytes	Cocci or chains	> 10%

*hpf* high power field (400× magnification)

A composite score of 1–2 represented normality. A score of 3–4 corresponded to slight aerobic vaginitis, a score of 5–6, moderate vaginitis, and a score above 6 (to max 10), severe aerobic vaginitis. In practice, a score of 8–10 was usually the same as so-called desquamative vaginitis, the most extreme form of aerobic vaginitis

### Patient data

After obtaining informed consent from each patient, patient data were collected from AV patients, including their age, AV infection status, density of vaginal bacteria, diversity of vaginal bacteria, bacteria that were predominant, levels of vaginal enzymes liking β-glucuronidase, leukocyte esterase, coagulase, and sialidase, and pH levels. Depending on AV infection status, those women who underwent routine gynecological examination and had simple AV infection were selected as the control group. The remaining patients allocated to the experimental group.

### Statistical analysis

Data were analyzed using SPSS software version 20. Categorical variables are presented as counts and percentages, and continuous variables as means ± standard deviation (SD). A chi-square or Fisher’s tests were used to compare categorical variables, and a *t*-test to compare quantitative variables in two groups. Multiple logistic regression analysis was used to calculate crude odds ratios (ORs) and 95% confidence intervals (CI). *P* < 0.05 was considered statistically significant. The confidence interval was set at 95%.

## Results

### Distribution of AV infections

In total, 284 sexually active patients who underwent routine gynecological examination had a diagnosis of AV and were recruited in strict accordance with the inclusion and exclusion criteria. All were aged 18–50 and menstruated regularly. Of the 284 patients with AV, 186 (65.49%) were found to have simple aerobic infection. The remaining 98 patients had mixed infection (34.51%). The most frequent infection combination was AV with BV (65/98, 66.33%), followed by AV with TV (13/98, 13.27%). Details of the distribution of infections are presented in Table 2.

**Table 2:** Infection status in the AV patients studied

Infection status	Age (means ± SD)	Case(s)	Percentage (%)
AV	44.58 ± 0.974	186	65.49%
AV + BV	40.85 ± 1.588	65	22.89%
AV + BV + CV	31	1	0.35%
AV + TV	38.38 ± 2.666	13	4.58%
AV + TV + BV	38.67 ± 2.042	12	4.23%
AV + VVC	36.50 ± 4.55	4	1.41%
AV + VVC + BV	31.00 ± 3.786	3	1.06%

### Proportions of mild, moderate and severe AV in simple and mixed AV

**Fig. 1:**
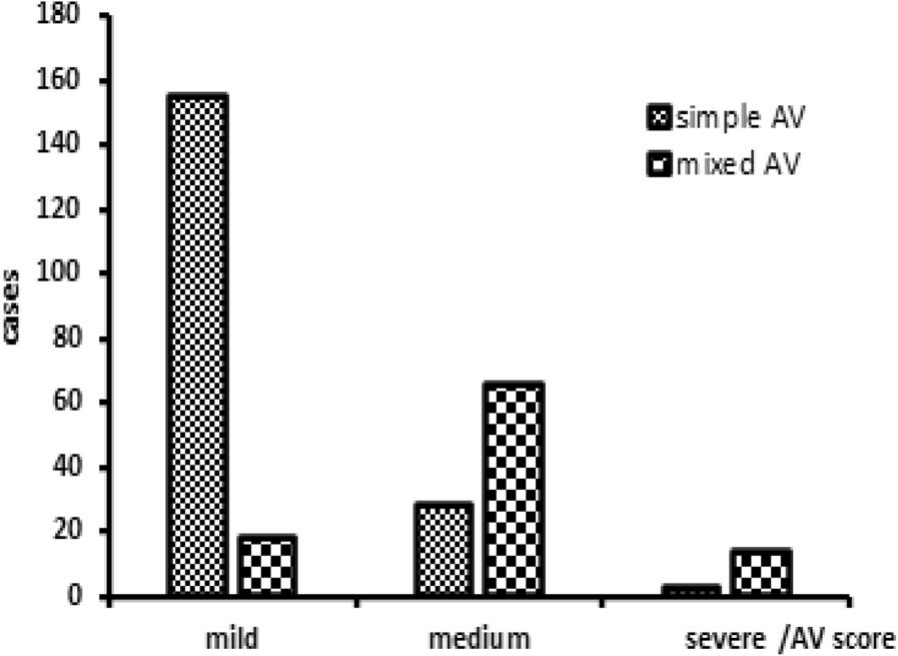
Proportion of mild, medium and severe AV in simple AV and mixed AV

According to the AV scores, mild AV infection was most common in simple AV infection, while moderate and severe AV were greater in mixed AV infection than in simple AV infection (Fig. 1).

### Distribution of ages of AV patients

**Fig. 2:**
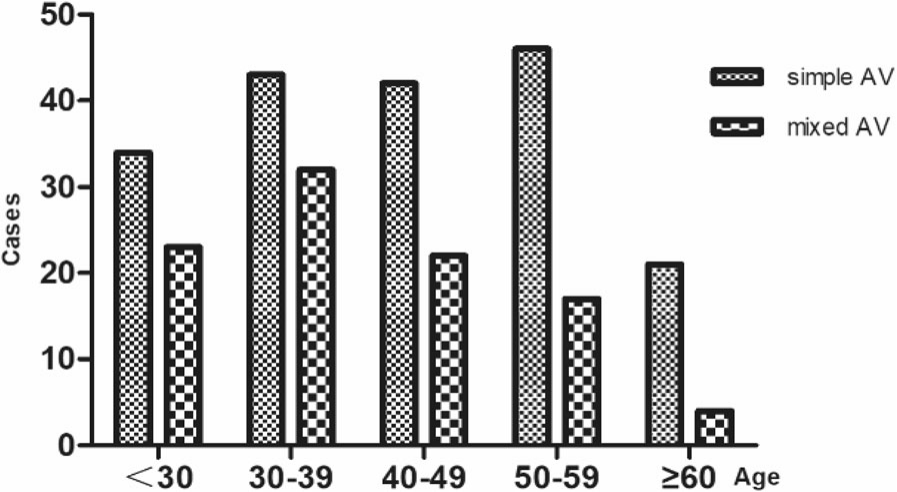
Age characteristics of AV patients

Simple AV infection was most common among patients aged 50–60, while mixed AV infection was more common among patients aged 30–40 (Fig. 2).

### Comparison of pure and mixed aerobic infections

Of the 284 AV patients, 175 cases were Gus(**β-glucuronidase)** positive (175/284; 61.62%), and 109 were Gus negative (109/284; 38.38%). A comparison between pure and mixed aerobic infections is shown in Table 3. The results indicate that those aged under 42 years were vulnerable to mixed AV (*P* = 0.003). Incidentally, the distribution of vaginal bacterial density, diversity of vaginal bacteria and predominance of bacteria were significantly different in simple AV and mixed AV patients (*P* < 0.01, respectively). From the aspect of functional detection, *β*-glucuronidase, coagulase and sialidase were significantly different between simple AV and mixed AV, respectively (*P* < 0.01, respectively), while no difference in hydrogen peroxide production (*P* = 0.655), leukocyte esterase (*P* = 0.428) and pH levels (*P* = 0.688) was observed between simple and mixed AV infections (Table 3).

**Table 3:** Comparison between pure and mixed aerobic infections (n[n%])

Clinical parameter	Total(***n*** = 284)	Simple AV infection (***n*** = 186)	Mixed AV infection (***n*** = 98)	***P***
**Age (mean ± SD, year)**	42.88 ± 0.766	44.58 ± 0.874	39.67 ± 1.167	**0.003**
**density of vaginal bacteria**				
II-III	272	185(99.46)	87(88.68)	**<0.001**
I or IV	12	1(0.54)	11(11.22)	
**diversity of vaginal bacteria**				**0.013**
II-III	264	178(95.70)	86(87.76)	
I or IV	20	8(4.30)	12(12.24)	
**predominant bacteria**				**0.002**
G^+^ coccus	175	145(77.76)	30(30.61)	
G^−^ bacilli	109	41(22.04)	68(69.39)	
**Hydrogen peroxide (H**_**2**_**O**_**2**_**)**				
Negative	1	1(0.54)	0(0)	0.207
Positive	283	185(99.46)	98(100)	
**β-Glucuronidase (Gus)**				**0.008**
Negative	256	175(94.09)	81(82.65)	
Positive	28	11(5.91)	17(17.35)	
**Leukocyte esterase (LE)**				0.428
Negative	3	3(1.61)	0(0)	
Positive	281	183(98.39)	98(100)	
**Coagulase**				**<0.001**
Negative	225	168(90.32)	57(58.16)	
Positive	59	18(9.68)	41(41.84)	
**Sialidase**				**<0.001**
Negative	247	174(93.55)	73(74.49)	
Positive	37	12(6.45)	25(25.51)	
**pH levels** **(mean ± SD)**	5.31 ± 0.014	5.31 ± 0.017	5.30 ± 0.024	0.688

*G+ coccus* gram positive coccus, *G- bacilli* gram negative bacilli

### Multivariate logistic regression analysis

Multivariate logistic regression analysis revealed that the density of vaginal bacteria (OR = 13.294, 95% CI = 5.869–30.115, *P* < 0.01) and bacteria that predominated (OR = 3.962, 95% CI = 1.785–7.984, *P* < 0.01) were risk factors for mixed AV. In addition, positive expression of coagulase (OR = 3.789, 95% CI = 1.798–7.984, *P* < 0.01) was also considered a risk factor of mixed AV (Table 4).

**Table 4:** Multivariate logistic regression analysis of risk factors for mixed AV

Parameters	β	S.E	Walds	***P***	OR (95% CI)
**Age(**mean ± SD)	−0.026	0.016	2.751	0.097	0.974(0.945–1.005)
**density of vaginal bacteria** (II-III vs. I or IV)	2.587	0.417	38.460	**<0.01**	**13.294(5.869–30.115)**
**diversity of vaginal bacteria** (II-III vs. I or IV)	0.545	0.530	1.059	0.303	1.725(0.611–4.870)
**predominant bacteria (**G^+^ coccus vs. G^−^ bacilli)	1.377	0.407	11.450	**<0.01**	**3.962(1.785–7.984)**
**β-Glucuronidase** (positive vs. negative)	0.064	0.334	0.037	0.847	1.066(0.554–2.052)
**Coagulase** (positive vs. negative)	1.332	0.380	12.269	**<0.01**	**3.789(1.798–7.984)**
**Sialidase** (positive vs. negative)	−0.229	0.459	0.424	0.515	0.742(0.302–1.824)

*OR* odds ratios, *CI* confidence interval

## Discussion

AV is a common form of vaginitis affecting millions of women that is distinguished from BV. A number of studies have found that the incidence of AV was approximately 11.77%, of which the incidence was approximately 13.08% in pregnancy and 4.34% in nonpregnant women [11]. Therefore, the present study aimed to analyze the differences between simple and mixed AV in order to assist in the diagnosis, treatment and management of patients. These results represent reference data for AV.

In the present study, 65.49% of cases were found to have a simple aerobic infection, and 34.51% had mixed infections. The results contradict the study of Wang (prevalence of simple AV: 38.67%, mixed AV: 61.33%) [17]. The reason for this may be that those women lived in a dry and conservative city. They will have sweated less, consistent with cleaner vulva and engaged in less hazardous sexual behavior. In addition, in the present study simple AV infection was most commonly observed in AV patients, focusing on elderly women in their 50s to 60s. Women younger than 42 years of age had a higher risk of mixed AV than more elderly women. The reason for mixed AV infection being more common in younger women may lie in their higher levels of estrogen, strong autoimmunity, and higher frequency of sexual activity. AV was found to be more commonly associated with BV (65/284, 22.89%), trichomonas vaginalis (13/284, 4.58%) and BV with TV (12/284, 4.41%). In a similar study, AV observed in 61.33% of patients was combined with other causes such as BV (33.67%), VVC (16.0%) or TV (5%) [17]. Although a number of researchers have found that AV infection is accompanied with other forms of vaginitis, such as candidiasis, trichomonas, and bacterial vaginitis, and presenting different clinical manifestations, the precise mechanism remains unclear [18–20]. The reason may be that the pathogenic bacteria in AV and BV habituate weak alkaline and inflammatory environments, an assumption that requires verification. From these results, medium and severe AV in mixed AV infection was more frequent than that in simple AV infection in terms of AV score, suggesting that vaginitis combined with AV infection was more associated with environments that were more inflamed and rich in a variety of bacteria in the vagina. However, no study has demonstrated different AV scores affecting different treatment strategies [21].

Compared with intestinal flora, vaginal flora in childbearing women consist of only 40 species of bacteria, with one variety of lactobacillus generally predominating, including *L. crispatus*, L. gasseri, L. iners, and L. jensenii, which produce lactic acid and build a vaginal immunological barrier [22]. The dominant bacteria in the vaginas of women of different races are significantly different, with lactobacillus in 80–90% of Asians and Caucasians, and in fewer than 60% of blacks and Hispanics [23]. The present study identified density and the species of predominant bacteria as independent risk factors for mixed AV infection, while no association between diversity of vaginal bacteria and mixed AV infection was found. However, if a change to predominant bacteria occurs, a loss of balance has an adverse consequence for the health of women. In addition, the diversity of vaginal bacteria also plays an important role in balance, but multivariate analysis in the present study suggests that diversity of vaginal bacteria possibly influences mixed AV but is not an independent risk factor.

Based on enzymatic activity, the results indicate that β-glucuronidase, coagulase and sialidase were positive in mixed AV patients. Wang demonstrated that in women positive for β-glucuronidase, coagulase and sialidase, AV was associated with *Escherichia coli* and B. streptococcus, with increased risk of cervical intraepithelial neoplasia (CIN) [17, 24]. Expression of the sialidase-encoding gene from Gardnerella vaginalis, was found to be significantly greater in persistent HPV infection, and may represent a microbial marker. The predominant pathogen of AV can decrease the beneficial effects of lactobacillus, causing inflammation, such as increased levels of IL-6, IL-8 and TNF-α, increasing the risk of HPV 16 infection which results in CIN or cervical cancer [25–27]. According to multivariate logistic regression analysis, only the effect of coagulase was found to be a risk factor in mixed AV patients. As is well known, coagulase positivity is a sign of *S. aureus* rather than other staphylococci [28]. The vaginal ecosystem has a diverse and complex microbiota that balances probiotic species and opportunistic pathogens [22]. So to treat AV infections, the results suggest that anti-staphylococcal drugs should be taken orally. In addition, previous studies indicate that lactoferrin may enhance the host’s vaginal innate immune system, regulating bacterial proliferation so that patients benefit from lactoferrin taken orally or as a pessary [29].

It is understood that the pH level was higher than 4.5 in AV patients, principally because microflora in AV were *Escherichia coli*, *Staphylococcus aureus*, coagulase-negative staphylococci etc., with lactobacillus levels lower in comparison [30]. In most patients with single VVC, the vaginal pH was normal, and in pure CV patients, the vaginal pH level was lower than 4.5 due to the high levels of lactobacillus, whereas in the majority of patients with BV, TV, AV, or AV mixed infections, the vaginal pH value was high (pH > 4.5) [19]. Unfortunately, the results indicate that pH levels in simple or mixed AV were not significantly different, but a recent study suggested that vaginal pH level had a significant association with CIN [31]. Therefore, due to the overlap between simple and mixed AV, it is difficult to draw firm conclusions for differentiating simple AV from mixed AV by pH level.

A limitation of this study is the lack of a negative control representing normal vaginal flora. The sample size was not large enough and the data describing the clinical characteristics, living conditions and hygiene of patients were inadequate, limiting the conclusions that can be drawn. Additionally, the study population was hospital-based and so bias cannot be avoided. Therefore, a systematic, clinical multicenter study is required to confirm these observations.

## Conclusions

In conclusion, aerobic vaginitis remains prevalent in simple infections, and the age of patients with simple AV was elder than those with mixed AV. In addition, the density of vaginal bacteria (I or IV), predominant bacteria and coagulase were high-risk factors for mixed AV infection. However, extensive epidemiological data and further studies are required to better understand the epidemiology of AV infection and the strategies aiming to treat simple and mixed AV infection.

## Data Availability

The data used can be obtained from the corresponding author upon reasonable request.

## Abbreviations

AV: Aerobic Vaginitis
BV: Bacterial Vaginitis
CV: Cytolytic Vaginosis
VVC: Vulvovaginal Candidiasis
TV: Trichomonas Vaginitis
H2O2: Hydrogen peroxide
LE: Leukocyte esterase
Gus: β-Glucuronidase
CIN: Cervical Intraepithelial Neoplasia
